# Unusual cause of abdominal pain: the disconnected tube after laparoscopic adjustable gastric banding

**DOI:** 10.11604/pamj.2017.28.184.13963

**Published:** 2017-10-30

**Authors:** Ahmed Bensaad, Christophe Fircket

**Affiliations:** 1 Hôpital Joseph Bracops, Réseau IRIS SUD, Bruxelles, Belgique

**Keywords:** Laparoscopic adjustable gastric banding, tube disconnection, abdominal pain

## Image in medicine

Laparoscopic adjustable gastric banding (LAGB) is a well recognised and valid option in the treatment of morbid obesity. The objective of this procedure is to restrict the overall food intake. The technique has the reputation of being associated with a low morbidity and almost no mortality. We report the case of a 31-year-old woman, with morbid obesity, who complained from a chronic abdominal pain, mostly localized in the hypogastirum and a failure to lose weight, 13 months after a LAGB. An abdominal X-ray was obtained showing the disconnected tubing from the port, with the separated tube slipping in the pelvis. The patient underwent a laparoscopic retrieval of the band at her will, which was uneventful. Tube disconnection must be considered in patient with history of LAGB, presenting with chronic abdominal pain. A reasonable length separating the port from entrance to the peritoneal cavity may diminish the incidence of this rare LAGB complication. Retrieval of the LapBand is not mandatory in all cases, since reconnecting or changing the port can help solve the problem.

**Figure 1:**
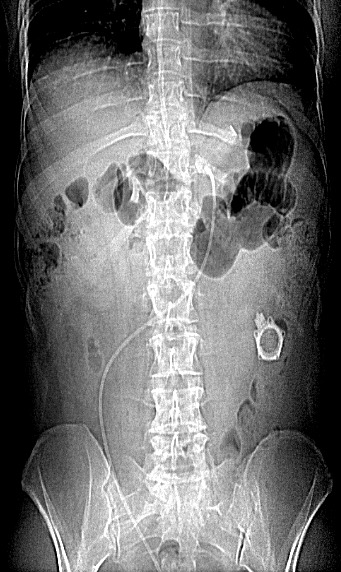
Abdominal X-ray showing disconnected tube from the port, with tube slipping in the pelvis

